# Exploring the Heat of Water Intrusion into a Metal–Organic Framework by Experiment and Simulation

**DOI:** 10.1021/acsami.3c15447

**Published:** 2024-01-23

**Authors:** Alexander R. Lowe, Piotr Ślęczkowski, Emre Arkan, Andrea Le Donne, Luis Bartolomé, Eder Amayuelas, Paweł Zajdel, Mirosław Chorążewski, Simone Meloni, Yaroslav Grosu

**Affiliations:** † Institute of Chemistry, University of Silesia, 40-006 Katowice, Poland; ‡ Dipartimento di Scienze Chimiche e Farmaceutiche Università Degli Studi di Ferrara, Via Luigi Borsari 46, Ferrara I-44121, Italy; § Centre for Cooperative Research on Alternative Energies (CIC EnergiGUNE), Basque Research and Technology Alliance (BRTA), Alava Technology Park, Albert Einstein 48, Vitoria-Gasteiz 01510, Spain; ∥ Institute of Physics, University of Silesia, 75 Pulku Piechoty 1, Chorzow 41-500, Poland

**Keywords:** heat, porous materials, temperature, simulation, water, calorimetry, nanoscale, pressure

## Abstract

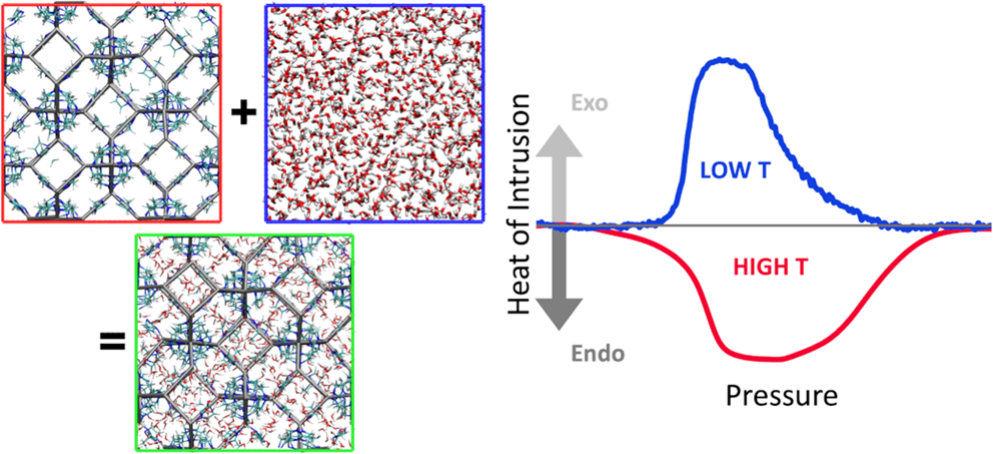

Wetting of a solid by a liquid is relevant for a broad range of natural and technological processes. This process is complex and involves the generation of heat, which is still poorly understood especially in nanoconfined systems. In this article, scanning transitiometry was used to measure and evaluate the pressure-driven heat of intrusion of water into solid ZIF-8 powder within the temperature range of 278.15–343.15 K. The conditions examined included the presence and absence of atmospheric gases, basic pH conditions, solid sample origins, and temperature. Simultaneously with these experiments, molecular dynamics simulations were conducted to elucidate the changing behavior of water as it enters into ZIF-8. The results are rationalized within a temperature-dependent thermodynamic cycle. This cycle describes the temperature-dependent process of ZIF-8 filling, heating, emptying, and cooling with respect to the change of internal energy of the cycle from the calculated change in the specific heat capacity of the system. At 298 K the experimental heat of intrusion per gram of ZIF-8 was found to be −10.8 ± 0.8 J·g^–1^. It increased by 19.2 J·g^–1^ with rising temperature to 343 K which is in a reasonable match with molecular dynamic simulations that predicted 16.1 J·g^–1^ rise. From these combined experiments, the role of confined water in heat of intrusion of ZIF-8 is further clarified.

## Introduction

The hydrophobic interactions between water and solids are fascinating since it is possible to exploit the generated nonwetting behavior for engineering purposes. Potential applications include self-cleaning surfaces,^1−3^ chromatography,^4,5^ energy-dissipation devices^6−8^ and energy-storage devices for either mechanical, electrical, or thermal energy storage.^6,9,10^ To study the process of combined mechanical and thermal energy storage along the interface of heterogeneous systems, certain specialized equipment are needed, examples, high-pressure lines and calorimeter cells can be inserted into a Calvet-type calorimeter,^8,11^ and from these humble beginnings, it has evolved to be a more sophisticated system, such as scanning transitiometers.^12,13^

To effectively study these heterogeneous systems, a standard liquid is required to fill the empty pores either by a passive or driven process. The filling process can be spontaneous, passively allowing a known liquid to penetrate into the empty cavities of the confining solid, and then initiate either melting–freezing transition of the liquid, to characterize the solid matrix, methods such as thermoporometry^14−16^ achieve this. A nonwetting case requires a significant amount of pressure to fill pores. This is achieved by mechanical methods, such as porosimetry.^17^ This is observed when the liquid (in this case, water) interacts with a hydrophobic solid with nanometer-sized pores.^18,19^ This is typical for some materials such as metal–organic frameworks, or grafted silica structures.

While thermal effects are important for the thermal management of industrial processes, they can be used to understand the state or nature of a more complex system, such as water moving into a confined environment. To simultaneously record both thermal energy and mechanical energy of this process to a porous hydrophobic material, pressure is applied to drive the water into the material. Scanning transitiometry, which applies a linear rate of pressurization has been utilized to examine the following solids: silicalite-1^20^ (otherwise known as MFI–OH^8^ and MFI-F^8^ modifications), ZIF-8,^12,21^ and Cu_2_(tebpz),^13,22^ and grafted silicas.^23^ Pressure perturbation calorimetric methods have also been used to investigate zeolites, such as silicalite-1^11,24^ (MFI^25^), CHA,^25^ and more recently ZIF-8.^26^ In this method, the pressure steps are initiated by controlled volume steps, which increase the system pressure. Regardless, the heat of intrusion into these materials is either endothermic or exothermic, with their recorded thermal heat effect determined by the solid’s topology^10,25^ or potentially the wettability of the system.^27^ Clearly, there is interest in understanding heat of wetting-drying of hydrophobic micropores; however, this subject remains challenging with respect to the continuum properties of water and down to its confinement at the nanoscale and its respective nanoscale phenomena.

For mesoporous materials, the specific heat of intrusion, *q* (J·g_ZIF-8_^–1^), can be rationalized through the “modified Kelvin equation” or Kelvin–Laplace law,^27^ defined as . Where *T* is the temperature (*K*), γ (mN·m^–1^) is the liquid–gas surface tension, θ (radian) is the liquid contact angle, and Ω is the specific surface area (m^2^·g^–1^) of the porous material. However, this relation breaks down as water becomes confined in smaller pore sizes, since the continuum description of the solid, liquid, and gas phases (and the capillary theory to represent their interfaces) beneath the Kelvin-Laplace equation becomes inadequate to describe these phenomena at a molecular level. For highly confined systems, the properties of individual phases and the interfaces between them need a molecular description. However, a complete theory is still lacking for this description.

Recent articles have demonstrated that the liquid intrusion of water into ZIF-8, at 25 °C, is either exothermic^26^ or endothermic^12^ demonstrating a considerable contradiction. In this paper, both calorimetric experiments and atomistic simulations were conducted on a ZIF-8 and water system to go further in understanding the heat of intrusion. Utilizing scanning transitiometery, the heat of intrusion for commercial and synthesized ZIF-8 was measured in the presence and absence of atmospheric gases, in acidic and basic solutions, and of production quality of ZIF-8. Simultaneously, molecular dynamics simulations were used to rationalize the temperature-dependent results with respect to the confinement issues between bulk and confined water.

## Materials and Methods

### Materials

Zinc nitrate hexahydrate (Zn(NO_3_)_2_·6H_2_O, >99.0%), 2-methylimidazole (Hmim, >98.0%), and methanol (MeOH, >99.5%) were purchased from Sigma-Aldrich. All chemicals were of analytical grade and used without any purification.

### ZIF-8 Samples

Two sources of ZIF-8 were used in this article. Commercial ZIF-8 was purchased from Aldrich as Basolite Z1200, LOT# STBG1590 V, CAS# 59061-53-9. The pore orifice size of ZIF-8 is 0.34 nm, with a surface area of approximately 1800 m^2^·g^–1^. Characterization of this material was done in the following articles by Lowe et al.^28^ and Zajdel et al.^29^

### Preparation of ZIF-8 (High Quality)

High quality ZIF-8 or ZIF-8 (HQ) was synthesized according to synthesis conditions reported by Zhang et al.,^30^ with slight modifications. Briefly, Zn(NO_3_)_2_·6H_2_O and Hmim were dissolved separately in 50 mL of MeOH solution each (solution A and solution B, respectively) in a 1:8 molar ratio. The solution B was poured into solution A under stirring at room temperature. The mixture was stirred for 90 min. Then, the solid product was collected from the dispersion by centrifugation (12,000 rpm, 35 min) and washed with MeOH three times. Finally, the obtained products were dried in air for 48 h.

### Water

Distilled water was prepared for each experiment and solution. Prior to each sample preparation degassed using a sonication at 40 °C for 30 min, then a 5 mbar pump was applied. The water was then used immediately afterward.

### Ammonia Solutions

A dilute solution of ammonia was prepared using 0.0492 g of ammonia solution [25 wt % purchased from Chempure (Series number 14/03/17)] in 156.2904 g of water. The final molarity was 0.0051 mol·L^–1^. The pH of the solution before was measured to be 10.3 after calibration with Certipur (Lot HC15455805) buffers, phthalate (4.01), phosphate (7.00), and borate (9.00) purchased from MERCK.

### Scanning Transitiometry

A BRG-tech scanning Transitiometer, located at the University of Silesia in Katowice, was used to simultaneously record the pressure–volume isotherms and thermograms between the pressure range of 5–55 MPa within the temperature range of 283–353 K. The recording process is simultaneous and applies a rate of pressurization of either 0.25 or 0.5 MPa·s^–1^. In these experiments, two calorimeter cells, one sample, and one reference, each rated for a maximum pressure of 200 MPa, were attached to a manifold that is connected to a single high-pressure line leading to high-pressure pump and stepper motor rated for a maximum pressure of 700 MPa. In this arrangement, the pressure in both cells changes simultaneously with the pressure applied by pump. The ZIF-8 sample cell, as described in the ZIF-8 Sample Preparation section, was inserted into the opening at the top of the measurement cell and rested on a support spring. Both calorimeter cells were filled with distilled and degassed water and then sealed at all points, with care taken to not add air bubbles into the cells. A scheme of the equipment can be found in the following reference, Lowe et al.^23^ By scanning with an applied increase/decrease of pressure, it is possible to record the differential heat effects of compression/decompression from the measurement and reference cells. The thermal effects of liquid intrusion/extrusion into a hydrophobic porous solid are then recorded as thermal events, where all associated processes proceed simultaneously. This is similar to a differential scanning calorimetry experiment related to protein denaturing, where the sample is dissolved in the reference liquid in the measuring cell, and the reference cell holds only the reference liquid. Upon the application of temperature, the calorimeter recorded the differential heat between the sample and reference cell. The change in apparent volume is calculated from the number of steps needed to push the volume of water through the high-pressure line by a high pressure piston. The steps are then calculated according to the change in the volume of liquid moved from the displaced piston.

A mass of ZIF-8 powder was weighed into a small stainless steel capsule with an outer diameter of 6 mm and an inner wall thickness of 0.5 mm. The bottom end of the tube was plugged with a ball of medical cotton wool compressed at one end and then weighed. The steel capsule was filled with the ZIF-8 powder and weighed again. The difference between both empty and full capsule provided the mass of ZIF-8 measured. The smallest mass of ZIF-8 was weighed at 48.6 ± 0.5 mg. A final cotton plug was placed within the tube to keep the powder in the tube. The sample tube was then treated in one of the following ways to determine the influence of gases and surround the sample in water.

#### Treatment 1

In a Schlenk flask, the air was evacuated to 5 mbar using a simple vacuum and held for 2 h under 90 °C bath oil to remove any water vapor. After the glass cooled, distilled/degassed water was allowed into the Schlenk flask to fill the empty space.

#### Treatment 2

Is a repeat of the outgassing treatment in the article by Zajdel et al.^31^ here, the Schlenk flask was connected to a high-vacuum Schlenk line manifold, then attached to the gas trap, which is submerged in liquid nitrogen to protect an Oerlikon Turbolab 80 vacuum pump. The recorded vacuum of 10^–5^ mbar was created to evacuate all the gas from the internal void space with the Schlenk tube submerged in a 90 °C silicon oil bath. After 4 h, the Schlenk flask was filled with distilled and degassed water surrounding the powder.

#### Treatment 3

The sample was not placed in any vacuum and was immersed into distilled and degassed water used directly.

### Theoretical Methods

**Figure 1:**
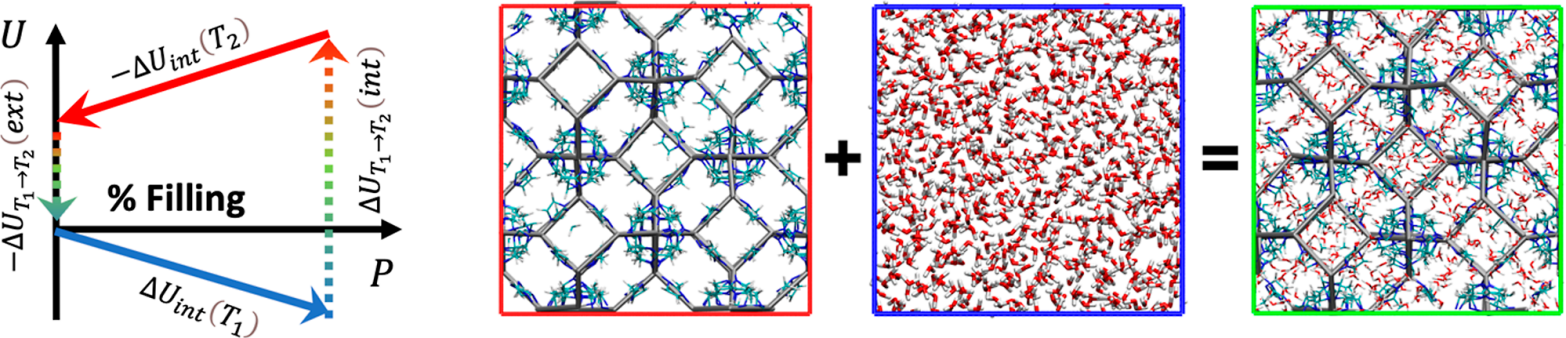
Thermodynamic cycle used to determine the temperature dependence of the heat of intrusion. The first step consists of the isothermal filling of ZIF-8 at *T*_1_. The filled system is warmed from *T*_1_ to *T*_2_. At *T*_2_, the system is extruded. Finally, the extruded system cooled back from *T*_2_ to *T*_1_, reaching the initial state. On the right, an image of the empty ZIF-8 solids (red), bulk water (blue), and fully intruded ZIF-8 (green) is reported.

To estimate the temperature dependence of the heat during the intrusion/extrusion process, from a theoretical point of view, the thermodynamic cycle of Figure 1 is introduced. The cycle consists of four steps, assumed to occur in quasi-equilibrium conditions, implying reversibility. The first step consists in an isothermal intrusion at temperature *T*_1_, with the corresponding change of internal energy denoted as Δ*U*_int_ (*T*_1_), followed by a isobaric heating from *T*_1_ to *T*_2,_ characterized by Δ*U*_*T*1→*T*2_(int), then the extrusion with Δ*U*_ext_(*T*_2_) = −Δ*U*_int_(*T*_2_) and, finally, an isobaric cooling from *T*_2_ to *T*_1_ with a variation of internal energy Δ*U*_*T*2→*T*1_(ext) = −Δ*U*_*T*1→*T*2_(ext). The overall variation in the internal energy along the cycle, corresponding to the sum of the four terms, is zero
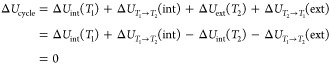
1

In the limit *T*_1_ → *T*_2_
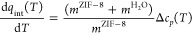
2where *q*_int_ (*T*) is the specific heat of intrusion, per unit mass of ZIF-8, at temperature *T*, and  is the mass-weighted average difference of the specific heat capacity of the intruded (*c*_*p*_^ZIF-8+H_2_O^) and extruded (*c*_*p*_^ZIF-8^, *c*_*p*_^H_2_O^) system. *m*^ZIF–8^ and *m*_H_2_O_ are the masses of ZIF-8 and intruded water, respectively. A detailed derivation of eq 2 is provided in the Supporting Information. Equation 2 relates the derivative of the intrusion heat on the temperature to the mass-weighted difference of the specific heat of the intruded and extruded system. Thus, the sign of Δ*c*_*p*_ determines the trend of *q*_int_ (*T*): a positive sign of the difference of the (mass weighted) specific heat capacity between the intruded and extruded states implies a growth of the intrusion heat with the temperature and vice versa.

From a quantitative point of view, eq 2 can be integrated to obtain *q*_int_ vs *T* provided that the value of Δ*c*_*p*_ (*T*) and the value of the specific heat of intrusion at a reference temperature (*T*_0_) are known
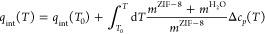
3

Δ*c*_*p*_ (*T*) is obtained from simulations, as explained in the following, and *q*_int_ (*T*_0_) = *q*_int_^exp^ (298 *K*) is the experimental specific heat of intrusion at 298 K. At this point the goal is to find a theoretical explanation of the trend of *q*_int_ vs *T*, rather than the absolute value of *q*_int_ at a given temperature, and eqs 1–3 provide a suitable theoretical framework to address this question.

The specific isobaric heat capacity of the three systems necessary to compute Δ*c*_*p*_ can be obtained from simulations by fluctuation of their enthalpy *H*:^32^*C*_*P*_ = (Δ*H*^2^)/(*k*_B_*T*^2^), with *k*_B_ the Boltzmann constant (“statistical mechanic” approach). Alternatively, the heat capacity can be computed from the (numerical) derivative of the enthalpy vs *T*: *C*_*P*_ = (∂*H*/∂*T*)_P_ (“thermodynamic” approach). In both cases, simulations are used to compute the ensemble averages corresponding to Δ*H*^2^ and *H*. In this latter case, it might be convenient to interpolate the *H* vs *T* suitable curve, first.

Here, the change in specific heat capacities is computed on a three periodic 2 × 2 × 2 ZIF-8 supercell computational sample, bulk water consisting of 900 molecules and the fully intruded ZIF-8. The simulations were carried out using LAMMPS package^33^ starting from an initial temperature of 280 K and reaching a final temperature equal to 360 K with 10 K steps in the constant number of particles, constant pressure (25 MPa, the intrusion pressure of ZIF-8), and constant temperature ensemble (*NPT* ensemble). Taking into account the reduction of the experimental intruded volume with temperature, the number of water molecules in the filled system decreases with temperature. At lower temperature, the 2 × 2 × 2 ZIF-8 supercell is filled with 640 water molecules; when the system is simulated at the highest temperature, the number of intruded water molecules is reduced to the final value of 592. The duration of the simulation of each system at each temperature is 20 ns divided in 5 ns of thermalization and 15 ns of the production run. The force field of Zheng et al.^34^ is used for modeling ZIF-8 potential energy combined with TIP4P/2005 model of water, a setup already successfully applied in the past.^35,36^ The fluctuations of pressure and temperature are controlled by Tobias-Klein-Martyna barostat^37^ applying a coupling constant of 1 ps and Nosè-Hoover chain thermostat with a coupling constant of 0.1 ps,^38^ respectively.

## Results

**Figure 2:**
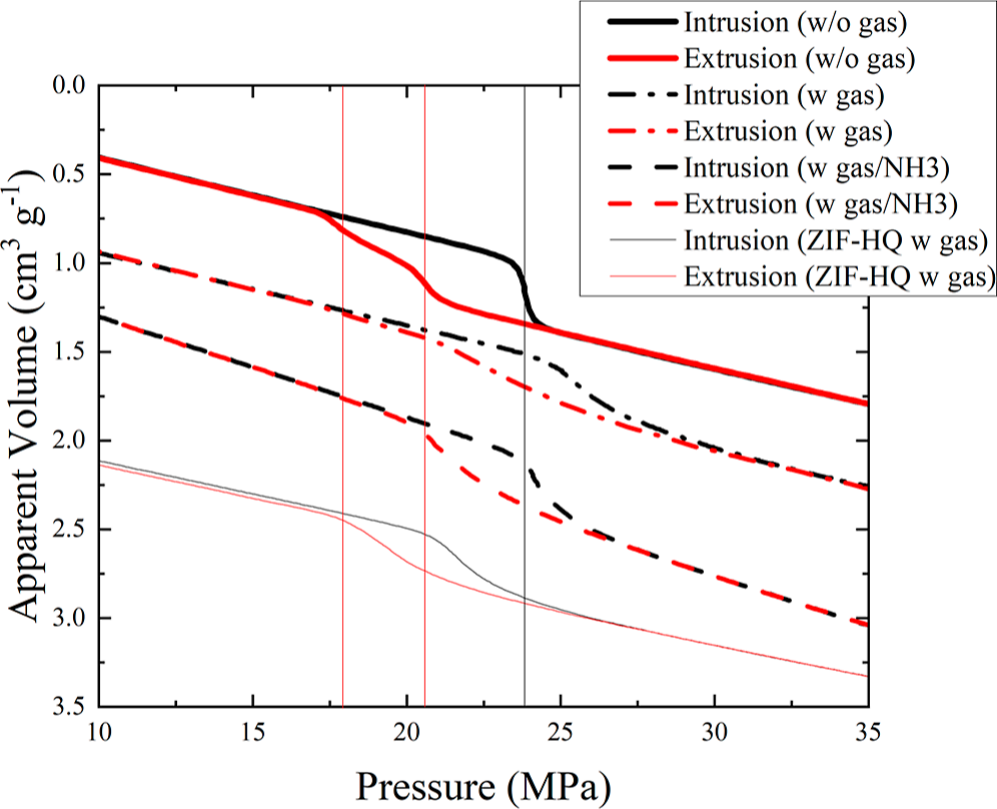
Liquid intrusion-extrusion (black-red) cycle of water entering ZIF-8, (outgassed at 10^–5^ mbar) at 298 K (thick solid lines), in the presence of gases (thick dot-dashed lines), in the presence of dilute ammonium hydroxide solution pH = 10.3 (dashed lines), and ZIF-8 (HQ) (thin solid lines) measured using scanning transitiometry. The vertical lines are guides to show the effects of gases and solutes on intrusion and extrusion pressures.

The PV-isotherm of ZIF-8 measured by using the scanning transitiometer is shown in Figure 2. As the pressure rises it reaches a point where the slope changes to a vertical position, indicating the liquid intrusion of water into the porous material. At the midpoint, the intrusion pressure is determined as 23.8 MPa. When the slope returns to its previous position the ZIF-8 is considered to be filled with liquid and the intrusion volume can be measured. The reverse direction from high pressure to low pressure leads to the extrusion process, where two slopes are observed at much lower pressures of 20.7 and 18.5 MPa. The complete extrusion of water from ZIF-8 shows that it can function as a spring or as a shock absorber depending on the dynamic conditions under multiple cycles. This has been demonstrated in the past in various articles.^12,26,39−42^ Additionally, when gas is present in the sample, the intrusions and extrusion isotherms do not show the level of detail with each process. The intrusion volumes are also slightly diminished by 0.05 cm^3^·g^–1^ with the two-step extrusion still partially visible. The addition of ammonia, which increased the alkalinity, restored some of the volume and sharpness of the isotherm, but the volume was still slightly smaller with lower intrusion\extrusion pressures. When ZIF–HQ was tested, the intrusion and extrusion pressures were smaller than the commercial sample and were not able to recover its initial intrusion volume upon subsequent cycles. The average values of intrusion/extrusion pressures and volume are presented in Figure S2 as a function of temperature. The comparison suggests that there is a mechanical difference between the commercial materials in the literature.^43^ It is during the intrusion process that the heat effects are studied in more detail and are the primary focus of this article.

**Figure 3:**
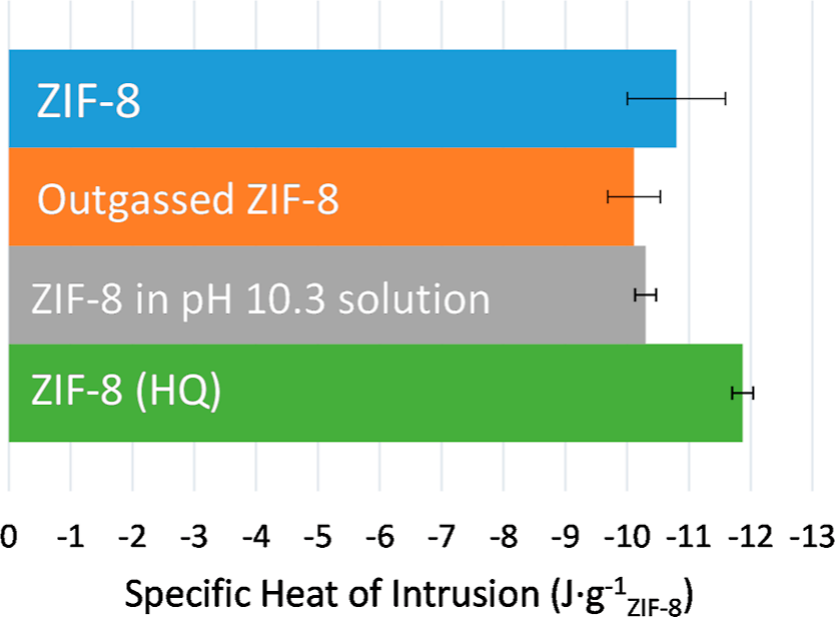
First bar is the average value of all measured specific heat of intrusion experiments of water into ZIF-8 at 298.15 K. All of the materials are not outgassed unless otherwise stated. Dilute ammonium hydroxide solution was used to adjust the pH of water to a pH of 10.3. ZIF-8 (HQ) represents the described synthesized sample.

The specific heat of intrusion (*q*_int_) is measured using the pressure scanning transitiometer under three different conditions with ZIF-8 provided from two different sources, which were either purchased from Sigma or prepared for this article. The results of the average heat of 3 cycles of ZIF-8 are seen in Figure 3.

These experiments were conducted at 298.15 K with PV-isotherms beginning at 5 MPa and completed at 55 MPa. The initial experiment demonstrates the results of Treatment 1, where the average exothermic heat is −10.8 ± 0.8 J·g_ZIF-8_^–1^. The second sample, where ZIF-8 was subjected to Treatment 2, was recorded to be −10.1 ± 0.4 J·g_ZIF-8_^–1^. The third sample was subjected to Treatment 3 with an ammonium hydroxide solution, and the measured heat was −10.3 ± 0.17 J·g_ZIF-8_^–1^. In this case, the experiment tests the effects of an increased pH on the heat of intrusion. The ZIF-8 (HQ) was subjected to Treatment 1 and produced an average heat of −11.87 ± 0.17 J·g_ZIF-8_^–1^. The results share the same sign as Astafan et al.^26^ who reported a lower heat of intrusion of −6.5 J·g_ZIF-8_^–1^. The measured results are different, both in sign and magnitude compared to the results of Grosu et al.^12^ who reported 4 J·g_ZIF-8_^–1^ (300 K).

**Figure 4:**
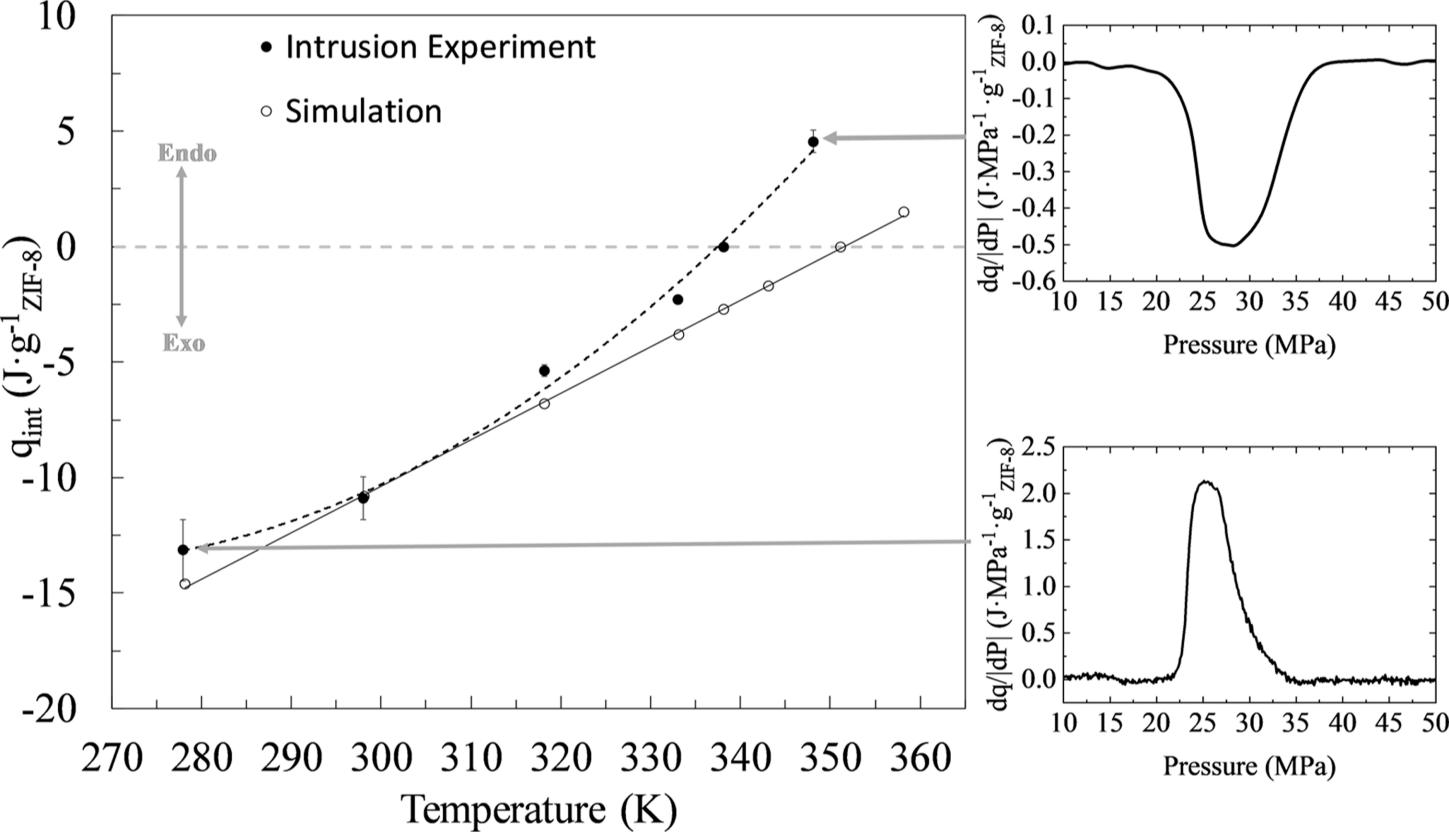
Specific heat of water intrusion (solid black) per gram of ZIF-8 from 278 to 343 K with dotted lines to guide the eye. The heat of intrusion of water with a higher pH = 10.3 is included into the average value, as it was shown to have a negligible effect. The hollow black circles and solid lines are simulated values. The top right panel demonstrates the endothermic heat of intrusion at 343 K and the bottom right panel demonstrates the exothermic heat of intrusion at 278 K.

In Figure 4, the averaged values of all the specific heats of intrusion experiments are plotted as a function of temperature. At the lowest temperature of 278.15 K the measured value is exothermic at −14.6 J·g_ZIF-8_^–1^, see the inset in Figure 4. As the temperature rises, the process becomes less exothermic until it reaches the transition point at 338 K. At 348 K, the process is completely endothermic reaching a value of 4.5 J·g_ZIF-8_^–1^, see inset in Figure 4. Simulation results of the intrusion cycle presented below show semiquantitative agreement with the experimental trends. The extrusion heats of the same cycles are presented in Figure S3. At temperatures higher than 333 K, the intrusion heat is no longer exothermic.

## Discussion

The heat of intrusion values collected from these experiments share the same thermodynamic sign as Astafan et al.,^26^ but are greater. The sign is opposite as compared to Grosu et al.,^12^ while the temperature trend is similar (Figure S3). The disagreement between these three sets of data suggests that heat generation upon water intrusion into nanopores is a complex phenomenon that can be affected by various conditions that are not yet identified. Thus, it is important to explore the factors that affect the heat of water intrusion into ZIF-8. These include basic pH conditions, presence of gases in the liquid and/or solid, and synthesis products. As can be seen from Figure 3, none of these experimental factors were able to identify a reason for the discrepancies. It was not possible to change the sign of the heat of intrusion by changing the value of any of these parameters within the range considered here. Though, a limited difference is observed when using ZIF-8 (HQ), which can be explained by the lack of observable impurities in the synthesis of the powder (Figure S4). Where the commercial sample presents extra peaks that belong to a secondary phase such as Zn(OH)(NO_3_)(H_2_O) and other unknown phases (Figure S4) generated from an nonoptimal synthesis protocol as seen in the articles from Zhang,^30^ Kida^44^ and Chen.^45^ However, ZIF-8 (HQ) compared to commercial ZIF-8 heat experiments only demonstrates a slightly greater absolute heat effect (11.87 ± 0.17 J·g_ZIF-8_^–1^ > 10.8 ± 0.8 J·g_ZIF-8_^–1^) supporting the idea that the final product of the synthesis method may influence the heat of intrusion. In the study from Astafan et al.^26^ they synthesized their own ZIF-8 and showed the heat of intrusion to be −6.5 J·g_ZIF-8_^–1^. The difference is too large to be fully explained by sample quality. The difference in the exothermic behavior between each sample may suggest that the structure and quality of the synthesized product can affect the heat values.

One issue, which has been brought up in the literature, is the degradation of ZIF-8 in water.^46,47^ Temperature has a significant effect on the degradation kinetics of ZIF-8. For experiments conducted below 318.15 K, ZIF-8 does not degrade significantly during sequential intrusion-extrusion cycling. Above this temperature, ZIF-8 begins to degrade after the third cycle leading to reduced heat of intrusion values with subsequent cycles. At the highest temperature of 348 K, the material is stable for two cycles with reproducible calorimetric signals generated. The heat of intrusion values of degraded samples were not included into the data presented in Figure 4.

When ZIF-8 is exposed to an acidic environment of either gas or liquid, the solid has been shown to chemically react and decompose.^48^ With this known, the effect of pH was studied using a dilute ammonia solution (0.0051 mol·L^–1^) of pH 10.3, to test if pH effects the heat of intrusion, since ammonia molecules possess a kinetic diameter of 0.326 nm compared to water’s which is 0.265 nm.^49^ While ammonia is bigger than water, both may pass through the ZIF-8 pore aperture of 0.34 nm.^40,50^ Both ammonia solution and water, seen in Figure 3, show similar heat of intrusion values within their respective standard deviation. Consequently, neither pH nor ammonia at this concentration had an effect on the recorded heat during the liquid intrusion.

The experiment regarding Treatment 2 was designed to remove as many atmospheric gases as possible to eliminate their effects during water intrusion into commercial ZIF-8. Under these conditions, the material does not show a great difference with the heat of intrusion results when compared to the other treatments and their effects (Figure 3). The result is similar to the ammonia solution, which resided within the standard deviation of the other experiments, which were exposed to Treatment 3. Therefore, gases do not play a substantial role in the heat of intrusion. From a cumulative point of view of the experiment results, the specific heat of intrusion into ZIF-8 is slightly affected by pH and the absence of atmospheric gases. The role of the synthesis and quality of ZIF-8 seems to be more noticeable but still has a limited effect.

The factor affecting heat of intrusion that was found to be consistent with a previous study is temperature.^12^ From our experiments, the heat of intrusion is exothermic below 337 K reducing in absolute value with temperature. Above 337 K it becomes endothermic, as seen in Figure 4. A similar temperature trend was previously reported for the heat of intrusion along the same temperature range with all of the experimental values displaying only endothermic behavior^12^ (Figure S3). To further understand the factors affecting the heat of intrusion, experiments were compared to computational methods (Figure 4), as described in the next section.

## Modeling Temperature Dependence

To begin elucidating the origin of the dependence of the heat of intrusion with respect to change in temperature, eq 2, was numerically integrated with the Δ*c*_*p*_ as determined from the computed isobaric specific heat capacities for the empty ZIF-8, bulk water, and water intruded ZIF-8 samples following the statistical mechanic approach (see eq 3). Based on the estimated change of specific heat capacity, the simulations predict a net change of specific heat of intrusion of 16.2 J·g_ZIF-8_^–1^ over a 70 K temperature range. This is in very good agreement with the 19.1 J·g_ZIF-8_^–1^ experimental specific heat of intrusion over the same temperature range. At higher temperatures, the experimental specific heat of intrusion has a pseudoparabolic trend, while the one obtained from eq 3 with Δ*c*_*p*_ computed from simulations shows a linear profile. The authors attribute the mismatch at higher temperatures to the sizable increase of vapor density in the ZIF-8 cages with *T*, which is not considered in these simulations where the contribution to Δ*c*_*p*_ arising from ZIF-8 has been computed from an empty framework. Here, the authors refrain from further improving these simulations to achieve a quantitative match, as the force fields that were used for these simulations are not optimized for the heterogeneous ZIF-8 and water systems. The objective here is to provide an explanation of the trend of the heat of intrusion with temperature, and the semiquantitative match is suitable to support the hypothesis that this depends on Δ*c*_*p*_.

Once established that the change of the specific heat capacity between intruded and extruded states is responsible for the trend of heat of intrusion on the temperature, the authors focused on the possible origin of the sign and absolute value of Δ*c*_*p*_. From previous simulations and experiments, it is estimated that the density of water confined within ZIF-8 cavities is approximated to be 0.7 kg·dm^–3^, which is much lower than the value of the bulk liquid.^35,41,51^ It is also noticed that the specific heat of bulk liquid water decreases with increasing density.^52^ Thus, the authors expect that the reduction of the density upon intrusion, from the bulk to the liquid-like confined value, is associated with an increase of heat capacity of bulk water (a positive Δ*c*_*p*_), which is consistent with simulation results.

Unfortunately, it is not possible to experimentally verify this qualitative argument, as it is not possible to measure the specific heat capacity of bulk water at an approximate density of 0.7 kg·dm^–3^. Under these intense tensile conditions, bulk water would cavitate. Simultaneously, neither experiments nor simulations permit the discrimination between the bulk-like and interface-like contributions to the heat capacity of confined water. Thus, it cannot be ruled out that interface-like effects contribute to the positive sign of Δ*c*_*p*_. In the future, the authors plan to assess the bulk-like vs interface-like effects by systematically studying ad hoc systems at different confined water densities and at various solid/liquid interface characteristics. For this, the authors will exploit the flexibility of simulations to explore artificial systems, systems that cannot be produced by experiment but can be created in computer simulations. This might lead to the identification of realistic systems with selected characteristics able to extract the individual bulk-like and interface-like effects on the temperature dependence of the heat of intrusion.

## Conclusions

The evolution of heat by water intrusion into a ZIF-8 is not a well-understood process, either experimentally or theoretically. From these experiments, the heat of intrusion is identified as exothermic at 298 K and becomes endothermic near 338 K. With respect to the values for the heat of intrusion, it appears that solutes such as atmospheric gases and dilute amounts of ammonia with a pH change do not play a significant role with its increase or decrease in value. However, origin does appear to play a role when comparing commercial ZIF-8 to ZIF-8 (HQ) where the latter presents a slightly larger heat of intrusion value.

The temperature-dependent heat values are determined primarily by the changes in density and specific heat capacity of the intruding liquid. This idea is supported by the developed theory, which defines the relationship between the change of water’s specific heat capacity upon intrusion and the dependence of the heat of intrusion with temperature. Using computer simulations, we estimated the values of this change of specific heat capacity were estimated. With only this parameter entered into the equation, from this, it became possible to predict a dependence of the heat of intrusion with respect to temperature with quantitative agreement with experiments. In particular, the positive trend of the heat of intrusion with temperature is due to a positive change in the specific heat capacity of the intruded system. This can be interpreted with respect to the pressure relationship of bulk water. When a rise in pressure increases the density but decreases the heat capacity. Intruded (confined) water decreases to approximately ∼0.7 kg·dm^–3^ , it follows that heat capacity increases in a similar direction in the intruded state leading to the observed and simulation heat of intrusion results.

## Supporting Information Available

Formulation of the thermodynamic cycle (PDF)
