# Supplementary material for: Exploring the Heat of Water Intrusion into a Metal–Organic Framework by Experiment and Simulation
Source: ACS Appl Mater Interfaces. 2024 Jan 23;16(4):5286–93. doi: 10.1021/acsami.3c15447 (PMC10835660; doi:10.1021/acsami.3c15447)
Supplement: Supplementary file 1 — am3c15447_si_001.pdf [file am3c15447_si_001.pdf]

SUPPORTING INFORMATION

Exploring the Heat of Water Intrusion into a Metal Organic Framework by Experiment and Simulation

Alexander R. Lowe,[1§] Piotr Ślęczkowski,[1#] Emre Arkan,[1] Andrea Le Donne,[2§] Luis Bartolomé,[3] Eder Amayuelas,[3] Paweł Zajdel,[4] Mirosław Chorażewski,[1*] Simone Meloni,[2*] Yaroslav Grosu[3*]*

[1] Institute of Chemistry, University of Silesia, 40-006 Katowice, Poland.

[2] Dipartimento di Scienze Chimiche e Farmaceutiche Università degli Studi di Ferrara, Via Luigi Borsari 46, I-44121, Ferrara, Italy.

[3] Centre for Cooperative Research on Alternative Energies (CIC EnergiGUNE), Basque Research and Technology Alliance (BRTA), Alava Technology Park, Albert Einstein 48, 01510 Vitoria-Gasteiz, Spain.

[4] Institute of Physics, University of Silesia, 75 Pulku Piechoty 1, 41-500 Chorzow, Poland

[*] To whom correspondence should be addressed; E-mail: alexander.lowe@us.edu.pl, miroslaw.chorazewski@us.edu.pl, simone.meloni@unife.it, ygrosu@cicenergigune.com

§A.R.L and A.L.D. contributed equally to this article.

1S. Theoretical predication of the temperature dependence of the heat flux

Starting from the thermodynamic cycle proposed in the main text (Figure 1), the procedure and the assumptions introduced to obtain the eq. 3 are reported here. First, we assume that the entire process (intrusion and subsequent extrusion) is performed under quasi-equilibrium conditions, i.e., any process is reversible; second, T_1 and T_2 are close enough that the intrusion and the extrusion pressures are the same, and so is the intruded volume at these temperatures. Under those hypotheses, the variation of the internal energy (U) for the cycle can be written as follows:

$$\Delta\Delta U = \Delta U_{\text{int}}(T_1) + \Delta U_{T_1 \to T_2}(int) - \Delta U_{\text{int}}(T_2) - \Delta U_{T_1 \to T_2}(ext) = 0 \qquad \text{(eq. S1)}$$

which can be recast into

$$\Delta U_{\text{int}}(T_1) - \Delta U_{int}(T_2) = -\left[\Delta U_{T_1 \to T_2}(int) - \Delta U_{T_1 \to T_2}(ext)\right]. \qquad \text{(eq. S2)}$$

Under the hypothesis that i) $P_{\text{int}}(T_1) \sim P_{\text{int}}(T_2)$ and ii) $\Delta V_{\text{int}}(T_1) \sim \Delta V_{\text{int}}(T_2)$ one gets

$$\Delta U_{\text{int}}(T_1) - \Delta U_{\text{int}}(T_2) =$$

$$[-\Delta V_{\text{int}} P_{\text{int}}(T_1) + Q_{\text{int}}(T_1)] - [-\Delta V_{\text{int}} P_{\text{int}}(T_2) + Q_{\text{int}}(T_2)] = Q_{\text{int}}(T_1) - Q_{\text{int}}(T_2) =$$

$$\left(m^{\text{ZIF}-8} + m^{\text{H}_2\text{O}}\right)\left[c_p^{\text{ZIF}-8+\text{H}_2\text{O}}(T_1) - \left(\frac{m^{\text{ZIF}-8}}{m^{\text{ZIF}-8} + m^{\text{H}_2\text{O}}} c_p^{\text{ZIF}-8}(T_2) + \frac{m^{\text{H}_2\text{O}}}{m^{\text{ZIF}-8} + m^{\text{H}_2\text{O}}} c_p^{\text{H}_2\text{O}}(T_2)\right)\right](T_1 - T_2) =$$

$$\left(m^{\text{ZIF}-8} + m^{\text{H}_2\text{O}}\right)\Delta c_p \Delta T \qquad \text{(eq. S3)}$$

where $\Delta c_p = \left[c_p^{\text{ZIF}-8+\text{H}_2\text{O}}(T_1) - \left(\frac{m^{\text{ZIF}-8}}{m^{\text{ZIF}-8} + m^{\text{H}_2\text{O}}} c_p^{\text{ZIF}-8}(T_2) + \frac{m^{\text{H}_2\text{O}}}{m^{\text{ZIF}-8} + m^{\text{H}_2\text{O}}} c_p^{\text{H}_2\text{O}}(T_2)\right)\right]$ and $\Delta T = T_1 - T_2$.

It is customary to report heat of intrusion per unit mass of the porous material, $q_{\text{int}} = Q_{\text{int}}/m^{\text{ZIF}-8}$. Thus, Eq. S3 can be cast into:

$$q_{\text{int}}(T_1) - q_{\text{int}}(T_2) = \frac{\left(m^{\text{ZIF}-8} + m^{\text{H}_2\text{O}}\right)}{m^{\text{ZIF}-8}} \Delta c_p \Delta T. \qquad \text{(eq. S4)}$$

In the limit $T_1 \rightarrow T_2$

$$\frac{dq_{\text{int}}(T)}{dT} = \frac{\left(m^{\text{ZIF}-8} + m^{\text{H}_2\text{O}}\right)}{m^{\text{ZIF}-8}} \Delta c_p(T). \qquad \text{(eq. S5)}$$

The specific heat capacity of the three systems necessary to compute Δc_p can be obtained from simulations either by fluctuation of their enthalpy H ($C_P = \Delta H^2/k_B T^2$, with k_B the Boltzmann constant, "statistical mechanic" approach) or by computing their enthalpy at various temperatures, and determining its derivative with respect to T ($(\partial H/\partial T)_P = H$, "thermodynamic" approach). In this latter case, it might be convenient to interpolate the H *vs* T suitable curve, first.

Figure S1 shows the enthalpy values of the three systems calculated in the range of temperature from 280 to 360 K. For all the three systems a linear fitting is reported. Applying this approach, one can notice that, for example the heat capacity of water, the c_p calculated is constant along the temperature range under consideration, which is not the realistic behavior.

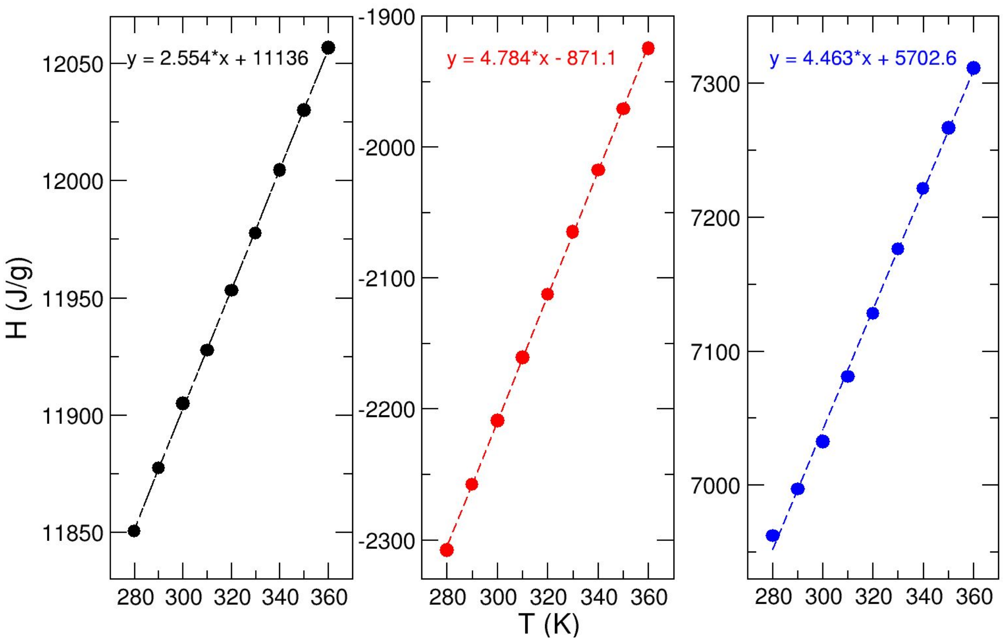

Figure S1. Enthalpy values for ZIF-8 (black), bulk water (red) and ZIF-8 filled by water (blue) at different temperatures. Enthalpy trends show a linear correlation with temperature (the dashed lines represent the linear fit).

In the literature [1-3], heat capacity obtained from classical molecular dynamics simulations is usually corrected for the so-called self-energy term, arising from the difference between the dipole moment of the model in bulk phase (μ_l) and that one of the gas phase (μ_g). The dipole moment-dependence of heat capacity on the aggregation phase, which changed by 1D or more [4], cannot be considered in a rigid water model with fixed charges. However, on the one end this term plays a lower role in our case, where we focus on the difference of heat capacity between bulk and confined water. Moreover, an analogous correction between these two phases of water requires the experimental dipole moment of confined water, which is not available. Hence, this correction is not included in our calculations.

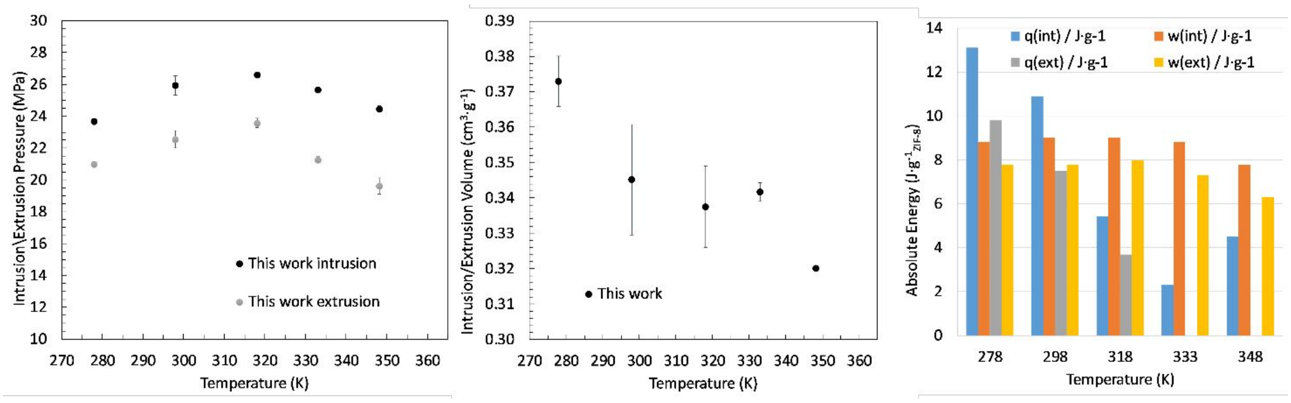

Figure S2: The figure on the left shows the average intrusion/extrusion pressure with increasing temperature up to 318K and decrease afterwards. The figure in the centre shows the average intrusion/extrusion volume of all experiments. With increasing temperature the intrusion volumes decrease with temperature. The experiments at 298.15 K possesses the largest standard uncertainty 0.02 cm^3 g^{-1}. The reason for these values is due to the difference in volume between experiments done in the presence of gases and absence of gases. The final figure on the right show the absolute mechanical ($w_{int/ext}$) and thermal ($q_{int/ext}$) energy ($J{\cdot}g^{-1}$) from intrusion and extrusion. Heats of extrusion were not recorded above 318K.

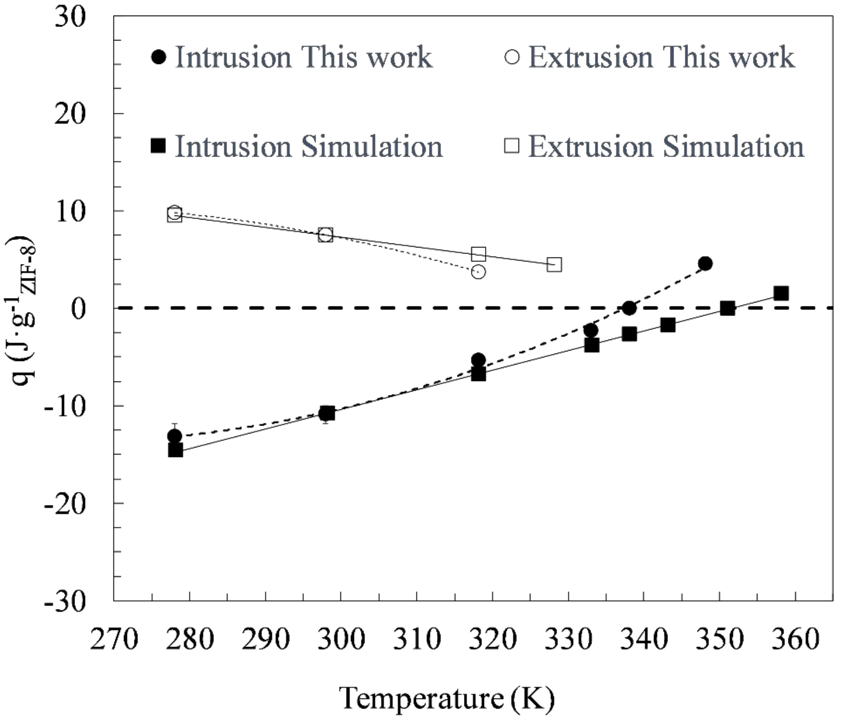

Figure S3: In black filled circles are the integral specific heats of intrusion from 278 to 348 K while the hollow black circles represent the integral specific heat of extrusion. The black squares represent the simulation results for intrusion (filled) and extrusion (hollow). The red and black dotted lines are for guiding the eye.

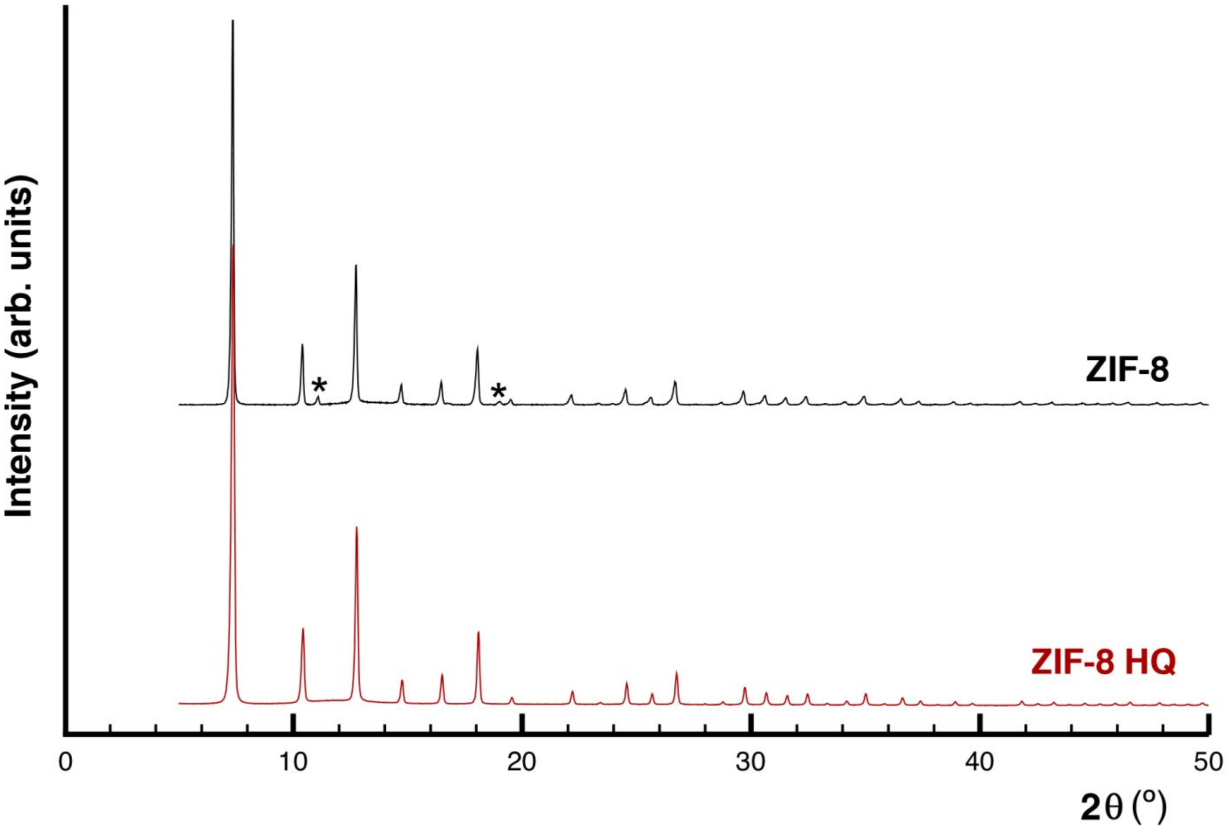

Figure S4. XRD for ZIF-8 and ZIF-8 HQ. Byproducts of the non-optimal synthesis protocol are highlighted with stars in the pattern of ZIF-8.

REFERENCES

1. Noya, E. G.; Menduiña, C.; Aragones, J. L. N.; Vega C. Equation of State, Thermal Expansion Coefficient, and Isothermal Compressibility for Ices Ih, II, III, V, and VI, as Obtained from Computer Simulation. *J. Phys. Chem. C* **2007**, 111, 43, 15877–15888.
2. Pi, H. L.; Aragones, J. L.; Vega, C; Noya, E. G.; Abascal, J. L. F.; Gonzalez, M. A.; McBride C. Anomalies in water as obtained from computer simulations of the TIP4P/2005 model: density maxima, and density, isothermal compressibility and heat capacity minima. *Mol. Phys.* **2009**, 107(4-6), 365–374.
3. Shvab, I.; Sadus, R. J. Atomistic water models: Aqueous thermodynamic properties from ambient to supercritical conditions. *Fluid Phase Equil.* **2016**, 407, 7–30.
4. Silvestrelli, P. L.; Parrinello, M. Water Molecule Dipole in the Gas and in the Liquid Phase. *Phys. Rev. Lett.* **1999**, 82, 3308.
